# Synthesis, Structures, and Sorption Properties of Two New Metal–Organic Frameworks Constructed by the Polycarboxylate Ligand Derived from Cyclotriphosphazene

**DOI:** 10.1021/acsomega.1c02492

**Published:** 2021-09-02

**Authors:** Jing-hua Han, Bing-qian Hu, Tangming Li, Hao Liang, Fan Yu, Qiang Zhao, Bao Li

**Affiliations:** † Key Laboratory of Optoelectronic Chemical Materials and Devices of Ministry of Education, School of Chemical and Environmental Engineering, Jianghan University, Wuhan, Hubei 430056, People’s Republic of China; ‡ School of Chemistry and Chemical Engineering, Hubei Key Laboratory of Bioinorganic Chemistry & Materia Medica, Huazhong University of Science and Technology, Wuhan, Hubei 430074, P. R. China

## Abstract

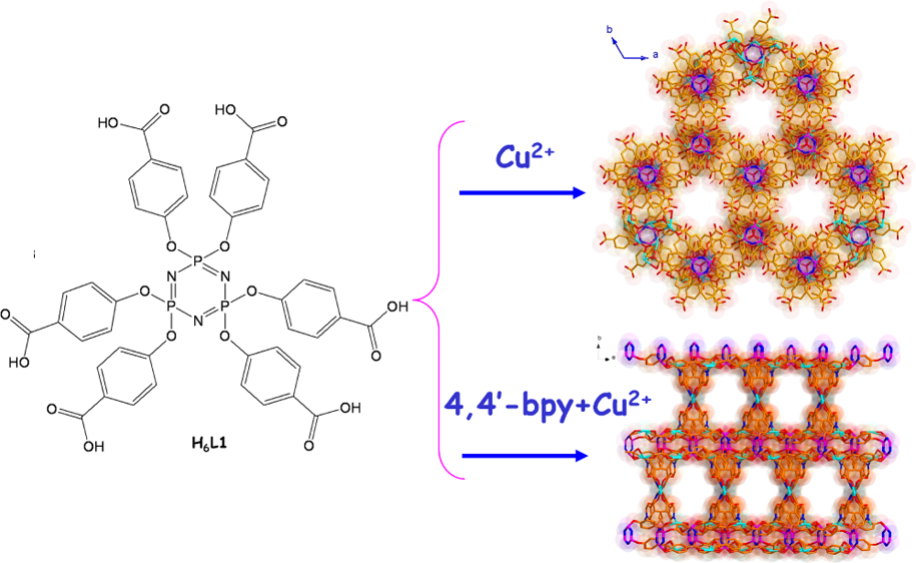

Solvothermal reactions of hexakis(4-carboxyphenoxy)cyclotriphospazene (H_6_L1) with copper ions in DMF/H_2_O produced one complex, {[Cu_6_(L1)_2_(OH)(H_2_O)_3_]·guest}_*n*_ (**1**), but with copper ions and auxiliary rigid 4,4-bipyridine (bpy) produced another new complex, namely, {[Cu_3_(L1)(bpy)(H_2_O)_6_]·guest}_*n*_ (**2**). These complexes had been characterized by IR spectroscopy, elemental analysis, and X-ray structural determination. **1** exhibits a 3D anionic structure with the binodal 4,8-connected network with Schläfli symbol {4^6^}_2_{4^9^·6^18^·8}, consisting of Cu_6_ clusters and L1 ligands. In contrast, complex **2** possesses a different 3D network with trinodal 3,4,6-c topology with Schläfli symbol {4·6^2^}_2_{4^2^·6^6^·8^5^·10^2^}{6^4^·8·10}. In these two complexes, the semirigid hexacarboxylate ligands adopt distinct conformations to connect metal ions/clusters, which must be ascribed to the addition of the auxiliary rigid ligand in reaction systems. In addition, gas absorption properties of **1** and **2** including CO_2_ and N_2_ were further investigated.

## Introduction

Great attention focused on metal–organic frameworks (MOFs) originated from not only the aesthetics network but also their versatile potential applications.^1−5^ The composition of MOFs definitely contains metal nodes (ions/clusters) usually named secondary building units (SBUs) and organic spacer (strut or bridging linker).^1−5^ The rational construction of MOFs depends on versatile conditions.^6−11^ Therefore, controllable building of MOFs still poses a difficult challenge. In terms of rational assembly, proper utilization of organic linkers would be of one facile method to tune the topologies.^12^ The configuration and connection mode of the selected ligands are the key points to determine the final structure of MOFs.^12^ For example, the utilization of versatile rigid polycarboxylate ligands would determine the variable MOFs.^13^ However, coordination assemblies containing highly connected flexible carboxylate ligands are relatively rare. The narrow investigation must be ascribed to the limited selection regions of aromatic scaffolds and the corresponding synthesis conditions. Compared to the reported highly connected carboxylates referred to aromatic templates, our group explored a hexacarboxylate ligand, hexakis(4-carboxyphenoxy)cyclotriphosphazene (H_6_L1), derived from a renowned inorganic heterocyclic ring to act as the central scaffold and used to construct new MOFs.

The utilization of this polycarboxylate ligand was mainly based on several considerations: first, the substituents at the three phosphorus sites can be readily varied by appropriate nucleophilic substitutions;^14^ second, the polycarboxylate derivation possesses variable configuration originated from the six twisted carboxylate arms, which is very facile to result in versatile architectures under controllable synthesis conditions; and third, MOFs constructed by this polycarboxylate ligand that exhibits highly and variously connected mode might be endowed high thermostability and unexpected topological framework. In addition, with the aid of the rigid N-containing ligands, the distinct topological framework would be also constructed compared to the mere reaction with various metal ions and this hexacarboxylate strut.^15^

With all the abovementioned considerations in mind, we tend to synthesize new MOFs assembled from metal ions and thus semirigid hexacarboxylate strut with/without the auxiliary rigid N-containing linker. Herein, we reported the synthesis and characterization of two MOFs, namely, {[Cu_6_(L1)_2_(OH) (H_2_O)_3_]·guest}_*n*_ (**1**) and {[Cu_3_(L1) (bpy)(H_2_O)_6_]·guest}_*n*_ (**2**). These new MOFs are characterized by elemental analysis, IR spectra, and X-ray crystallography.

## Results and Discussion

### Synthesis of **1** and **2**

**Figure 1:**
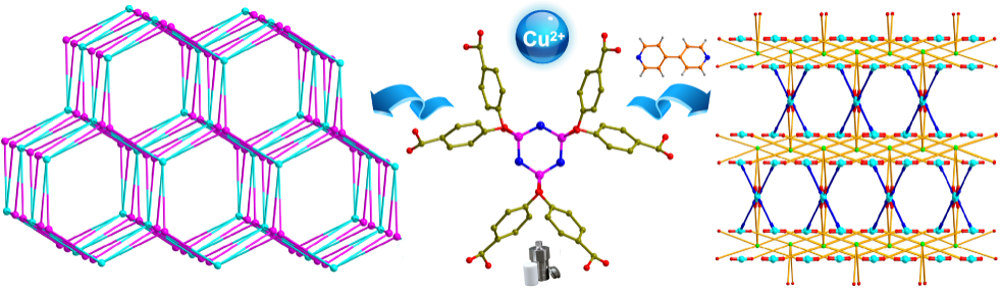
Reaction route of **1** and **2**.

Compound **1** was prepared under solvothermal conditions by heating an acid mixture of Cu(NO_3_)_2_·3H_2_O and H_6_L1 with a mole ratio of 10:1 in DMF/H_2_O (v/v, 1 mL/0.2 mL) at 80 °C for 2 days. In contrast, compound **2** was prepared under solvothermal conditions by heating an identical mixture and solvent with the additional bpy at 100 °C for 5 days. Crystal data of **1** and **2** were gathered and are shown in Table S1. The relative qualified X-ray diffraction result of **1** after repeated attempts was given due to the weak diffraction intensity and small size of the crystalline sample. However, the clear structure could be observed after careful dissolution, which could also be verified by the XRD and EA results (Figure 1).

### Crystal Structure of **1**

**Figure 2:**
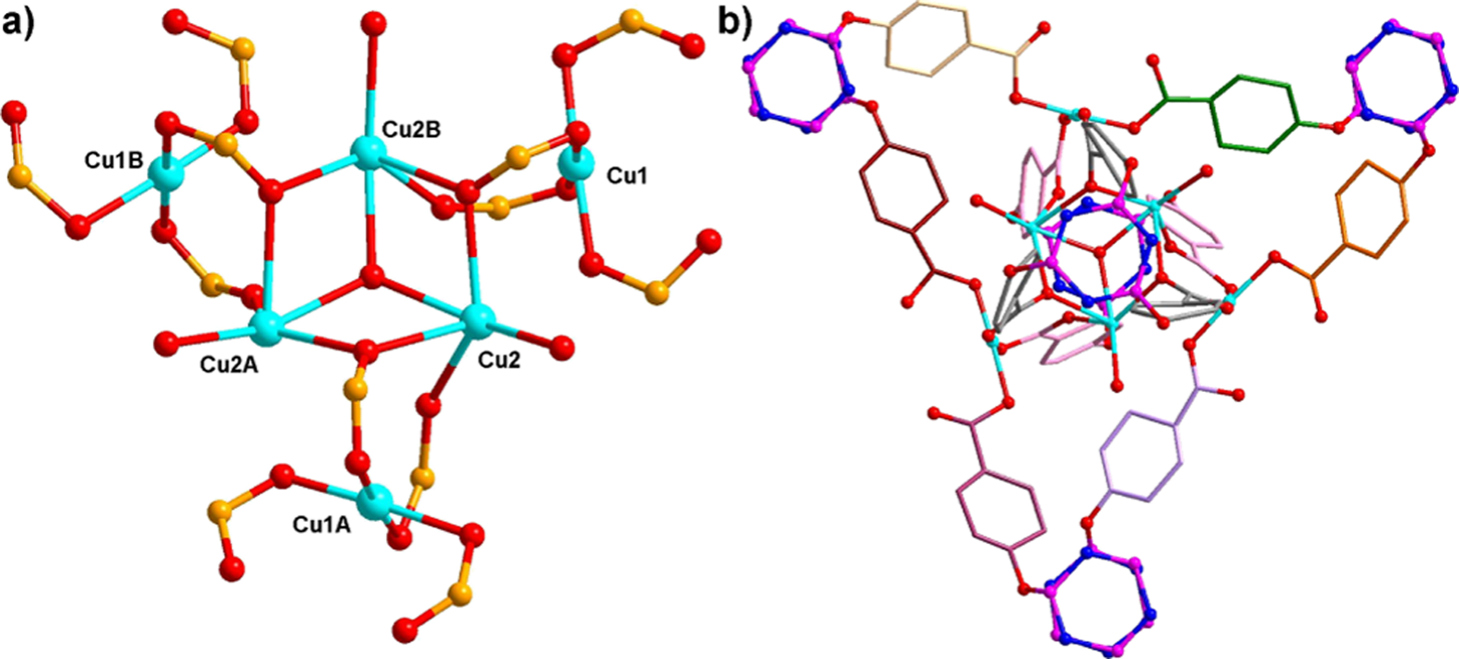
Perspective of the hexanuclear copper SBU (a) and eight-connected mode of Cu_6_ SBU (each colorful carbon atoms represents one pendant of the L1 ligand) (b). Symmetric code: A, −*x* + *y*, −*x* – 1, *z* and B, −*y* – 1, *x* – *y* – 1, *z*.

**Figure 3:**
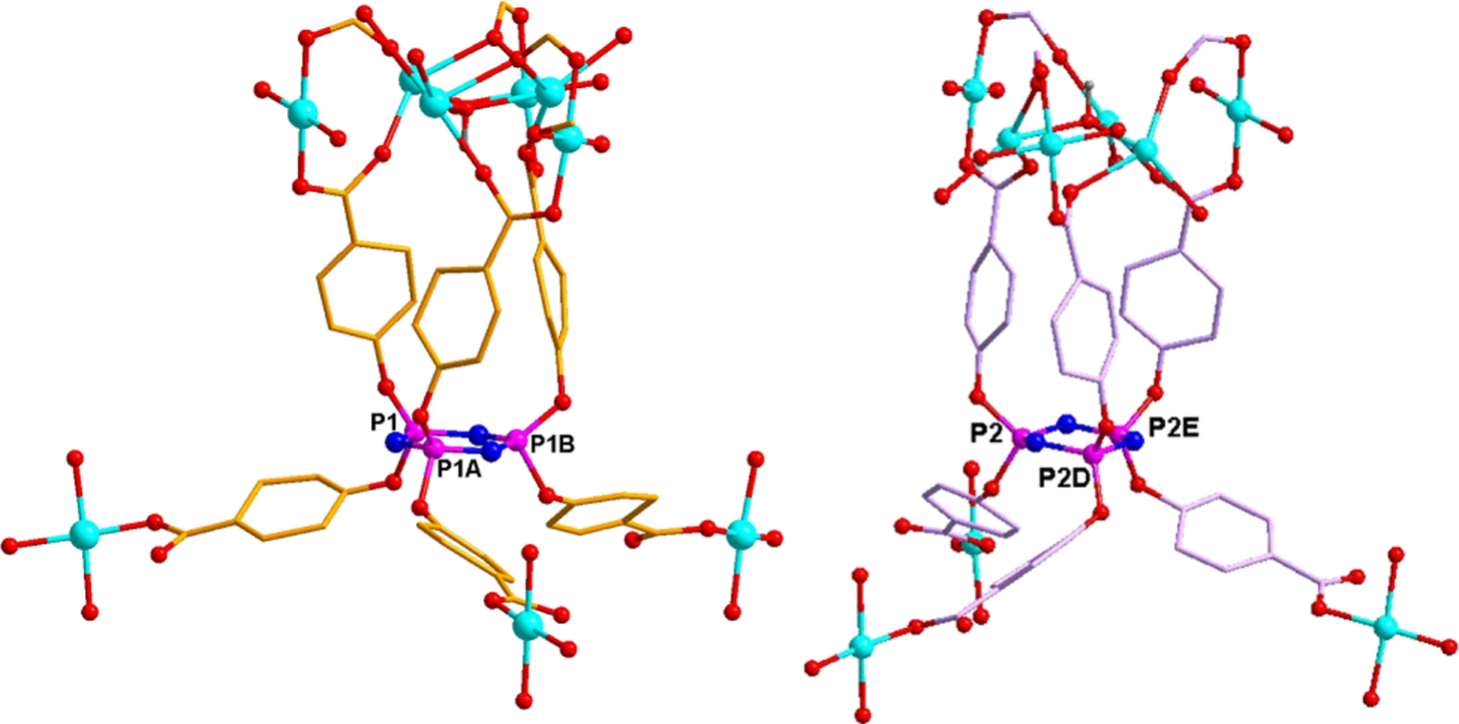
Connection modes of two types of four-connected hexacarboxylate ligands in **1**. For clarity, all hydrogen atoms are omitted. Symmetric code: A, −*x* + *y* + 1, −*x*, *z*; B, −*y*, *x* – *y* – 1, *z*; D, −*x* + *y*, −*x* – 1, *z*; and E, −*y* – 1, *x* – *y* – 1, *z*.

A single-crystal X-ray diffraction study performed on compound **1** reveals that it is a high symmetric three-dimensional (3D) framework, crystallized in trigonal space group *P*3̅*c*1. The asymmetric unit of **1** contains two copper ions, one coordinated water, and two types of 1/3 ligand and hydroxyl (Figure S3). The Cu(1) atom adapts square planar geometry, coordinated by four oxygen atoms from four carboxylates in three types of the connection mode: mono-dentate, syn–syn μ_2_^b^ bridging, and syn-syn-anti μ_3_-η^2^:η^1^ tridentate coordination mode, as shown in Figure 2a. In contrast, the Cu(2) atom adapts a slightly distorted trigonal bipyramid with the τ_5_ parameter of 0.3, whose equatorial vertices comprised three O atoms separately from different carboxyl of L1 in two coordination modes: syn–syn μ_2_^b^ bridging and syn–syn–anti μ_3_-η^2^:η^1^ tridentate coordination mode, and the axial positions are occupied by two oxygen atoms separately from one coordinated aqua molecule and μ_3_-hydroxyl group (Figure 3). Cu(1) and Cu(2)–O bond lengths range from 1.920(8) to 2.430(7) Å, similar to the typical Cu^II^–O bond lengths. Three Cu(2) atoms are interlinked by the oxygen atom of the μ_3_-hydroxyl group, which are further connected reciprocally by one oxygen atom of the tri-dentate carboxyl to form the tri-nuclear core. Moreover, three Cu(1) atoms are interconnected to Cu(2) atoms by two kinds of carboxylate groups (three syn–syn carboxyl from one hexacarboxylate ligand and three tridentate carboxyl from another one) to form the hexanuclear copper SBU (Figure 2a). Three coordinated aqua molecules on Cu(2) atoms are decorated on the surface of the tri-nuclear core to fulfill the coordination environment. Thus, planar metal-cluster-based SBUs are extended by eight hexacarboxylate ligands through six mono-dentate carboxylate groups of different ligands at the parallel plane of Cu6 SBU and six bridging carboxyls separately from two hexacarboxylate ligands along the perpendicular direction of Cu6 SBU, as shown in Figure 2b.

**Figure 4:**
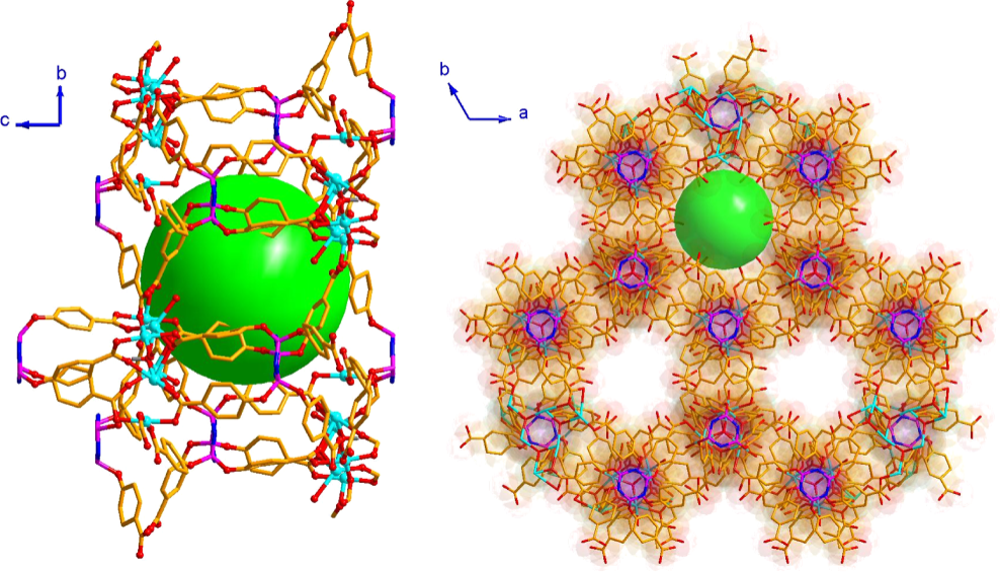
Partial view of cavity and the 3D crystal structure of **1**, along with the 1D channels.

**Figure 5:**
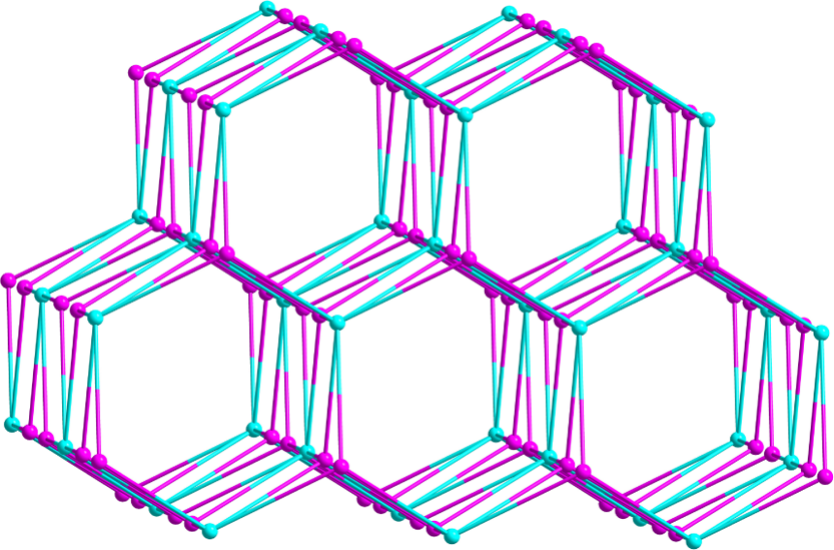
Perspective view of the 4,8T11 topological structure of **1** (the blue and purple balls represent the eight-connected Cu_6_ SBUs and four-connected L1 ligands, respectively).

In **1**, there are two distinct connection modes of hexacarboxylate ligands, which are connecting four hexanuclear copper SBUs into an infinite 3D anionic open framework. Large cavities with a diameter of 15.37 × 9.06 Å were embedded in the whole structure and arranged along the *c* axis (Figure 4), which might be filled with the counter-cation. The uncoordinated oxygen atoms of mono-dentate carboxyls and coordinated aqua molecules point toward the center of the pores, which might play an important role in the field of gas sorption or catalysis. The solvent-accessible volume in the dehydrated structure of **1** is about 45.4%, calculated by *PLATON* routine. Considering the copper SBUs and hexacarboxylate ligands as eight- and four-connecting nodes, respectively, **1** topologically possesses a 4,8-connected 2-nodal net with stoichiometry (4-c)_2_(8-c) and the point (Schläfli) symbol {4^6^}_2_{4^9^·6^18^·8} calculated with *TOPOS* software (Figure 5),^16^ which has been reported as 4,8T11 topological type. The connection of hexanuclear clusters and hexacarboxylate ligands is responsible for the generation of different topologies compared to the structure consisted of paddle-wheel Cu_2_ cluster and hexacarboxylate ligands.

### Crystal Structure of **2**

**Figure 6:**
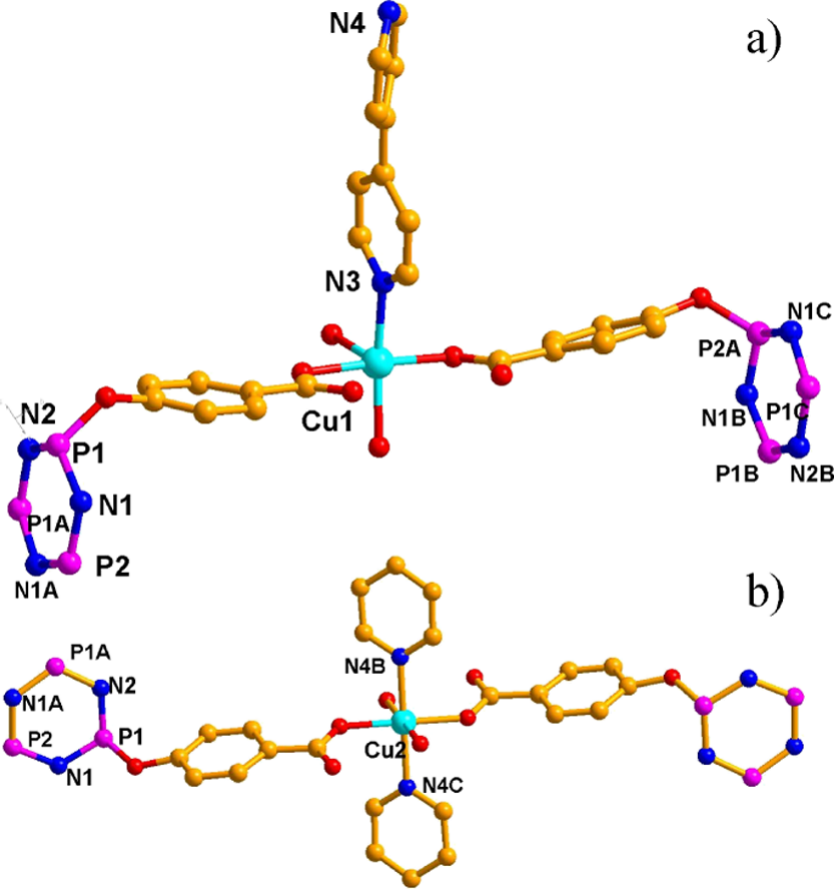
Coordination environment of copper ions in **2**. For clarity, all hydrogen atoms are omitted. Symmetric code: (a) A, −*x* + 1, *y*, −*z* + 1/2; B, −*x* + 2, −*y*, −*z* + 1; C, *x* + 1, −*y*, *z* + 1/2 and (b) A, −*x* + 1, *y*, *z* + 1/2; B, *x* – 1/2, – *y* + 1/2, *z* – 1/2; C, −*x* + 1, *y*, −*z* + 3/2.

The reaction of the same copper salt and hexacarboxylate ligands with the auxiliary N-containing pillar, bpy, has produced another MOF **2** which also exhibit a three-dimensional (3D) framework. **2** crystallizes in monoclinic space group *C*2/*c*, along with one copper ion named Cu(1), one bpy pillar, three coordinated aqua molecules, and one-half of copper ion named Cu(2) and ligand in an asymmetric unit, shown in Figure S4. The Cu(1) atom adapts a square-pyramidal with a τ_5_ parameter of 0.07. The basal plane is constructed by one pyridyl N (N3) atoms of bpy and three oxygen atoms separately from two mono-dentate carboxyls and one coordinated water, while the apical position is occupied by another coordinated aqua molecule, as shown in Figure 6a. In contrast, the Cu(2) atom adapts a slightly distorted octahedral environment. The equatorial vertices consist of two O atoms separately from different mono-dentate carboxyl of L1 and two nitrogen atoms (N3) from two byp ligands, and the axial positions are occupied by two oxygen atoms from two coordinated aqua molecules, as shown in Figure 6b. The Cu–O and–N bond lengths range from 1.945(3) and 2.452(4) Å, similar to the many previous reported Cu^II^–O and–N bond lengths.

**Figure 7:**
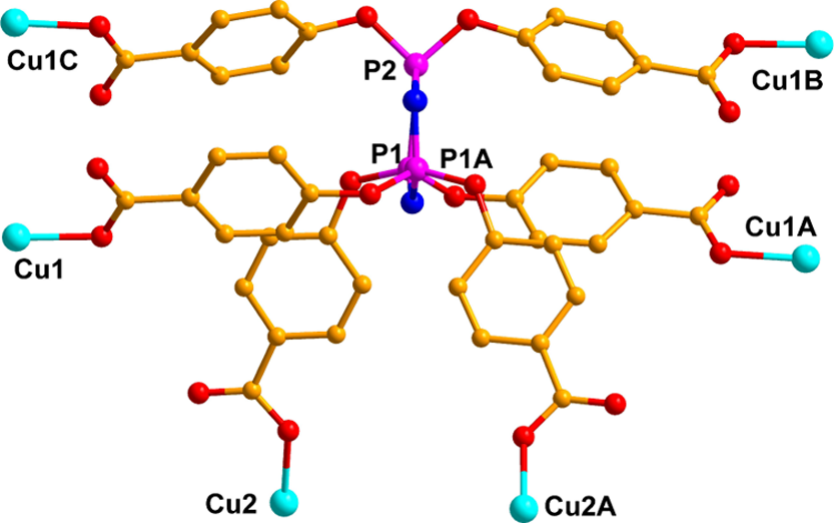
Coordination environments of the six-connected polycarboxylate ligand. For clarity, all hydrogen atoms are omitted. Symmetric code: A, −*x* + 1, *y*, −*z* + 1/2; B, *x* – 1, −*y*, *z* – 1/2; and C, −*x* + 2, −*y*, −*z* + 1.

**Figure 8:**
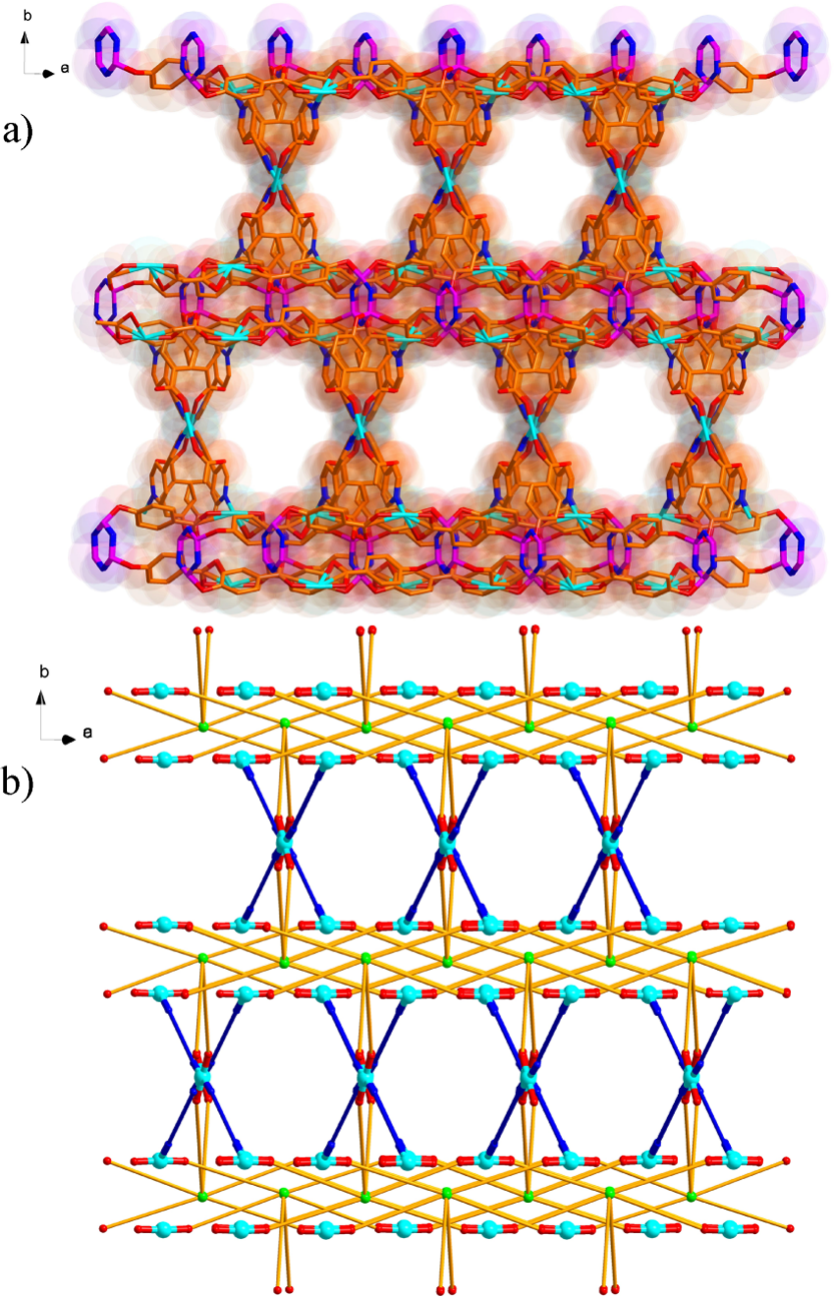
(a) Partial view of the 3D structure of **2** showing the 1D channels along the crystallographic *c*-axis direction and (b) view of the topological structure of **2** (blue and green balls represent the four-connected Cu ions and six-connected L1 ligands, respectively; the deep-blue lines represent the linear linker, byp).

In **2**, the center-symmetric hexacarboxylate ligands connect six copper ions by its mono-dentate carboxylate group, as shown in Figure 7. Each extended ligand connects four copper ions. As such, all of the hexacarboxylate ligands serve as six-connected bridges to link different metallic nodes into an infinite 3D crystal structure, which are further stabilized by the rigid bpy pillars located along the *b* axial direction, shown in Figure 8. 1D channels with a diameter of 16.25 × 12.21 Å were embedded in the whole structure and arranged along the *c* axis (Figure 7). The coordinated aqua molecules on copper ions also point toward the center of pores. The solvent-accessible volume in **2** is about 42.0%, calculated by *PLATON* routine. Considering the two kinds of copper ions, hexacarboxylate ligands, and bpy as three-, four-, six-, and two-connecting nodes, respectively, the overall structure of **2** topologically possesses a 3,4,6-connected 4-nodal net with stoichiometry (3-c)_2_(4-c) (6-c) and the Schläfli symbol {4·6^2^}_2_{4^2^·6^6^·8^5^·10^2^}{6^4^·8·10} calculated with *TOPOS* software (Figure 8).^16^ Thus, the topological type has not been found in the database according to the routine of *TOPOS*.

### Thermal Stability

**Figure 9:**
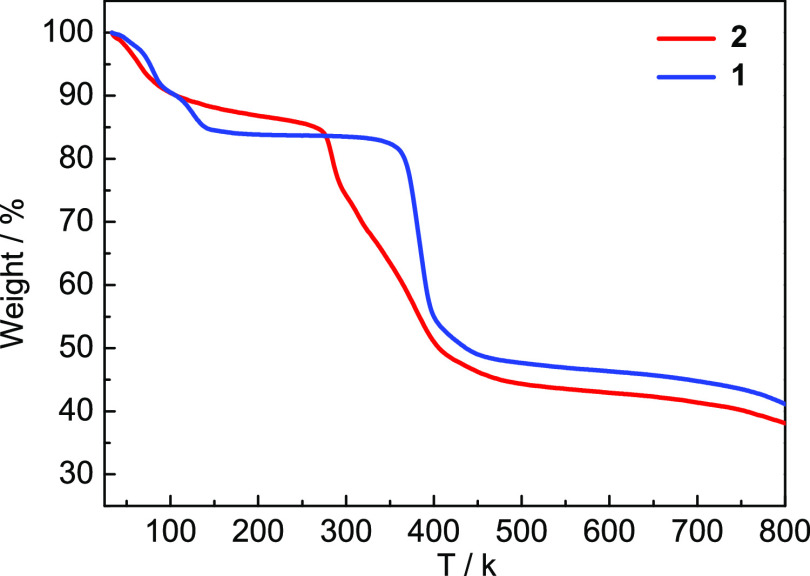
TGA curves of **1**–**2**.

Thermal stability of **1**–**2** had been further examined by thermogravimetric analyses (TGAs) by the utilization of crystalline samples under a N_2_ atmosphere (Figure 9). Observed from the TGA curve, **1** undergoes dehydration before 150 °C and decomposition around 360 °C. For **2**, the weight loss from 30 to 130 °C should be ascribed to the loss of lattice water molecules, and the decomposition began around 300 °C. The high connection mode of the hexanuclear clusters must be the main reason for the higher thermal stability of **1** compared to **2**.

### Sorption Properties

**Figure 10:**
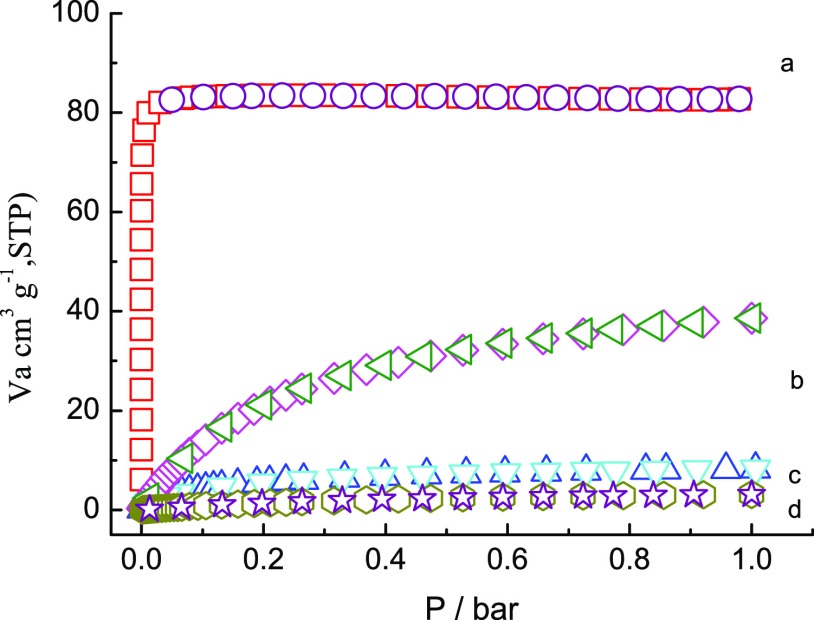
Sorption isotherms for **1** and **2**: (a) N_2_ (1) at 77 K; (b) CO_2_ (1) at 273 K; (C) N_2_ (2) at 77 K; and (D) CO_2_ (2) at 273 K.

In order to validate the pores of the porous samples of **1** and **2**, the gas sorption experiments of hydrogen and CO_2_ have been carried out. The desolvated samples were produced via immersing in methanol and acetone. Then, the solid sample was vacuum-dried at room temperature and 120 °C overnight, respectively. Observed from the adsorption curves, the typical type-I adsorption isotherms with no significant hysteresis between sorption and desorption traces had been presented, indicating the microporous materials for **1**. The adsorption amount of N_2_ at 77 K is 87 cm^3^/g, along with the calculated Brunauer–Emmett–Teller (BET) and Langmuir surface areas of 275 and 367 m^2^/g. Comparably, the desolvated samples of **1** continuously exhibit the low-pressure CO_2_ adsorption via volumetric gas adsorption measurements, illustrating the fully reversible adsorption behavior. The approximate 29 cm^3^/g CO_2_ uptake had been observed under 273 K and 1 bar (Figure 10). The XRD pattern of samples after the adsorption experiments is similar to the fresh sample and simulated pattern (Figure S1), which also illustrates the stable framework for **1**. However, the porous structure of 2 collapsed during the procession of desolvent, confirmed by the XRD spectra and gas absorption experimental measurements.

## Conclusions

In summary, two MOFs using the flexible hexacarboxylate ligand derived from a renowned inorganic heterocyclic cyclotriphosphazene have been successfully synthesized and structurally characterized. The structural results show that using the flexible ligand would be of a good approach to produce diversity in final structures. **1** exhibits a 3D anionic structure incorporating with hexanuclear Cu^II^ SBUs and two kinds of hexacarboxylate ligands, while **2** has a 3D topology with a 1D channel resided in the structure. The highly connected hexanuclear SUBs must be the main reason for the higher thermal stability compared **1** to **2**, as well as the stable desolvated structure of **1** for the application of gas sorption.

## Experimental Section

### Synthesis of {[Cu_6_(L1)_2_(OH) (H_2_O)_3_]·Guest}_*n*_ (**1**)

A mixture of Cu(NO_3_)_3_·3H_2_O (30 mg) and H_6_L1 (10 mg) was dissolved in 15 mL of DMF/H_2_O (1:2, v/v), and then, the pH value was adjusted to 2–3. The final mixture was heated at 80 °C under autogenous pressure for 48 h in 10 Parr Teflon-lined stainless-steel vessels and then cooled to room temperature. The resulting solution was allowed to stand undisturbedly from which square-like crystals were obtained. The crystals were collected together, washed with the mother liquid, and dried under ambient conditions. Yield of the reaction was ca. 41% based on H_6_L1. Anal. Calcd for dehydrated C_84_H_55_Cu_6_N_6_O_40_P_6_: C, 42.83%, H, 2.35%, N, 3.57%; found C, 42.03%, H, 3.02%, N, 4.07%. For fresh sample: C, 41.89%, H, 3.51%, N, 4.36%. Calculated with EA and TGA data, the whole formula of **1** should be {(C_2_H_6_NH)·[Cu_6_(L1)_2_(OH) (H_2_O)_3_]·DMF·(H_2_O)_5_}_*n*_. IR (KBr, cm^–1^): 3481, 1603, 1543, 1420, 1384, 1210, 1159, 966, 790.

### Synthesis of {[Cu_3_(L1) (bpy) (H_2_O)_6_]·Guest}_*n*_ (**2**)

The synthesis process was very similar to **1** except adding 4,4-bipyridine 20 mg in the reaction system, adjusting the pH range located in 4–5 for 120 h. Square-like crystals were directly obtained, and crystals were filtered off, washed with the mother liquid, and dried under ambient conditions. Yield of the reaction was ca. 23% based on H_6_L1. Calcd for dehydrated C_62_H_52_Cu_3_N_7_O_24_P_3_: C, 47.65%, H, 3.35%, N, 6.27%; found C, 46.98%, H, 3.03%, N, 6.87%. For the fresh sample: C, 45.65%, H, 3.05%, N, 4.74%. Calculated with EA and TGA data, the whole formula of **2** should be {[Cu_3_(L1) (bpy) (H_2_O)_6_]·(H_2_O)_8_}_*n*_. IR (KBr, cm^–1^): 3418, 1604, 1540, 1420, 1384, 1211, 1159, 967, 790.

## Supporting Information Available

Detailed experimental sections, thermal spectra, and XRD spectrum of **1**–**2** (PDF).
(CIF)
(CIF)

## Supplementary Material

ao1c02492_si_001.pdf

ao1c02492_si_002.cif

ao1c02492_si_003.cif
