# Supplementary material for: Synthesis, Structures, and Sorption Properties of Two New Metal–Organic Frameworks Constructed by the Polycarboxylate Ligand Derived from Cyclotriphosphazene
Source: ACS Omega. 2021 Sep 2;6(36):23110–6. doi: 10.1021/acsomega.1c02492 (PMC8444217; doi:10.1021/acsomega.1c02492)
Supplement: Supplementary file 1 — ao1c02492_si_001.pdf [file ao1c02492_si_001.pdf]

Synthesis, Structures and Sorption properties of Two New Metal-Organic Frameworks Constructed by Polycarboxylate Ligand Derived from cyclotriphosphazene

Jing-hua Han,[a] Bing-qian Hu,[a] Tangming Li,[a] Hao Liang[a], Fan Yu, [a,b]* Qiang Zhao,[b] Bao Li[b,*]

[a]*Key Laboratory of Optoelectronic Chemical Materials and Devices of Ministry of Education, School of Chemical and Environmental Engineering, Jianghan University, Wuhan, Hubei 430056, People's Republic of China. Email:yufan0714@163.com*；bSchool of Chemistry and Chemical Engineering, Huazhong University of Science and Technology, Wuhan, Hubei 430074, P. R. China.*Email:libao@hust.edu.cn*

Experimental sections:

Materials and General Methods. All reagents were purchased from commercial sources and were used without further purification. FT–IR spectra were recorded as KBr pellets with an Equinox 55 FT–IR spectrophotometer (4000–400 cm^{-1}). Thermal gravimetric analyses (TGA) were performed under N_2 atmosphere (100 ml/min) with a heating rate of 4°C/min between ambient temperature and 500°C using a Pyris1 thermogravimetric analyzer. Powder X-ray diffraction (PXRD) data were collected over the 2θ range 5~60° using a X'Pert PRO automated diffractometer at room temperature, with a step size of 0.02° in 2θ angle. The hexa-carboxylate ligand was synthesized accodring to the literature(Dalton Trans., 2013, 42, 2588).

Synthesis of {[$Cu_6(L1)_2(OH)(H_2O)_3$]·Guest}$_n$ (1). A mixture of $Cu(NO_3)_3\cdot 3H_2O$ (30 mg), H_6L1 (10 mg) was dissolved in 15 mL of DMF/H_2O (1:2, v/v), and then pH value was adjusted to 2-3. The final mixture was heated at 80℃ under autogenous pressure for 48 hours in 10 Parr Teflon-lined stainless steel vessels, and then cooled to room temperature. The resulting solution was standed undisturbedly from which square-like crystals were obtained. The crystals were collected together, washed with mother liquid, and dried under ambient conditions. Yield of the reaction was ca. 41% based on H_6L1. Anal. Calcd for dyhydrated $C_{84}H_{55}Cu_6N_6O_{40}P_6$: C, 42.83%, H, 2.35%, N, 3.57%; found C, 42.03%, H, 3.02%, N, 4.07%. For fresh sample: C, 41.89%, H, 3.51%, N, 4.36%. Calculated with EA and TGA data, the whole formula of **1** should be {(C_2H_6NH)·[$Cu_6(L1)_2(OH)(H_2O)_3$]·DMF·$(H_2O)_5$}$_n$. IR (KBr, cm^{-1}): 3481, 1603, 1543, 1420, 1384, 1210, 1159, 966, 790.

Synthesis of {[$Cu_3(L1)(bpy)(H_2O)_6$]·Guest}$_n$ (2). The synthesis process was very similar to **1** except adding 4,4-bipyridine 20mg in the reaction system, adjusting the pH range located in 4~5 and for 120 hours. Square-like crystals were directly obtained, and crystals were filtered off, washed with mother liquid, and dried under ambient conditions. Yield of the reaction was ca. 23% based on H_6L1. Calcd for dyhydrated $C_{62}H_{52}Cu_3N_7O_{24}P_3$: C, 47.65%, H, 3.35%, N, 6.27%; found C,

46.98%, H, 3.03%, N, 6.87%. For fresh sample: C, 45.65%, H, 3.05%, N, 4.74%. Calculated with EA and TGA data, the whole formula of **2** should be $\{[Cu_3(L1)(bpy)(H_2O)_6]\cdot(H_2O)_8\}_n$. IR (KBr, cm^{-1}): 3418, 1604, 1540, 1420, 1384, 1211, 1159, 967, 790.

X-Ray Structural Determination. Suitable single crystals were selected and mounted onto the end of a thin glass fiber. X-ray intensity data were measured on a Bruker SMART APEX CCD II based diffractometer with graphite-monochromated Cu for **1** and Mo Kα radiation for **2**. The structure was solved by direct methods and refined by full-matrix least squares using the *SHELXTL* crystallographic software package.[1] All the non-hydrogen atoms were refined anisotropically. The hydrogen atoms were generated theoretically onto the specific atoms and refined isotropically with fixed thermal factors. The hydrogen atoms of the coordinated water molecules were not located for compounds **1**-**2**. In compound **1-2**, because of disorder, thermal parameters of disordered atoms atoms were restrained. In addition, due to the highly disordered solvent molecules in compounds **1–2**, the PLATON/SQUEEZE routine[2] was employed to calculate the diffraction contribution from the solvent molecules, and thereby to produce a set of solvent-free diffraction intensities. The size of the crystalline sample of **1** was too small to give the strong diffraction intensity, and subsequently gave the unqualified crystal data with high R1 and WR2 values. Not unusual for large flexible complexes might be responsible for the CheckCIF alerts of **1**. The final formula was derived from crystallographic data combined with elemental and thermogravimetric analyses data. Details of the crystal parameters, data collection and refinements for complexes **1–2** are summarized in Table S1. CCDC 2083131 (**1**) and CCDC 2083132 (**2**) contain the supplementary crystallographic data for this paper. The data can be obtained free of charge at www.ccdc.cam.ac.uk/conts/retrieving.html.

Reference：

1 G. M. Sheldrick, *SHELXTL-PLUS, Crystal Structure Analysis Package*; Bruker Analytical X-Ray; Madison, WI, USA, **1997**.

2. PLATON program: A. L. Spek, *Acta Crystallogr. Sect. A*, **1990**, *46*, 194.

Low pressure gas sorption measurements: Low-pressure N_2 adsorption measurements (up to 1 bar) were performed on Micromeritics ASAP 2020 M+C surface area and pore size analyzer. About 200 mg of methanol solvent-exchanged samples were activated at 90 °C for 12 hours by using the "outgas" function of the surface area analyzer. Helium was used for the estimation of the dead volume, assuming that it is not adsorbed at any of the studied temperatures. To provide high accuracy and precision in determining P/P_0, the saturation pressure P0 was measured throughout the N_2 analyses by means of a dedicated saturation pressure transducer, which allowed us to monitor the vapor pressure for each data point. A part of the N_2 sorption isotherm in the P/P_0 range 0. 01–0.1 was fitted to the BET equation to estimate the BET surface area and the Langmuir surface area calculation was performed using all data points. The pore size distribution (PSD) was obtained from the DFT model in the Micromeritics ASAP2020 software package (assuming slit pore geometry) based on the N_2 sorption at 77 K.

Table S1 crystal data of 1-2

Compound reference	compound1	compound2
Chemical formula	$C_{84}H_{55}Cu_6N_6O_{40}P_6$	$C_{62}H_{52}Cu_3N_7O_{24}P_3$
Formula Mass	2355.40	1562.57
Crystal system	Trigonal	Monoclinic
a/Å	18.2270(2)	16.6520(12)
b/Å	18.2270(2)	33.684(2)
c/Å	49.8038(15)	16.5376(12)
α/°	90.00	90.00
β/°	90.00	90.4980(10)
γ/°	120.00	90.00
Unit cell volume/$Å^3$	14329.2(5)	9275.7(12)
Temperature/K	293(2)	273(2)
Space group	*P*3*c*1	*C*2/*c*
Z	4	4
No. of reflections measured	5064	15283
R_{int}	0.0732	0.0461
Final R_1 values ($I > 2\sigma(I)$)	0.1017	0.0467
Final $wR(F^2)$ values ($I > 2\sigma(I)$)	0.2905	0.1400
Final R_1 values (all data)	0.1820	0.0586
Final $wR(F^2)$ values (all data)	0.3234	0.1469

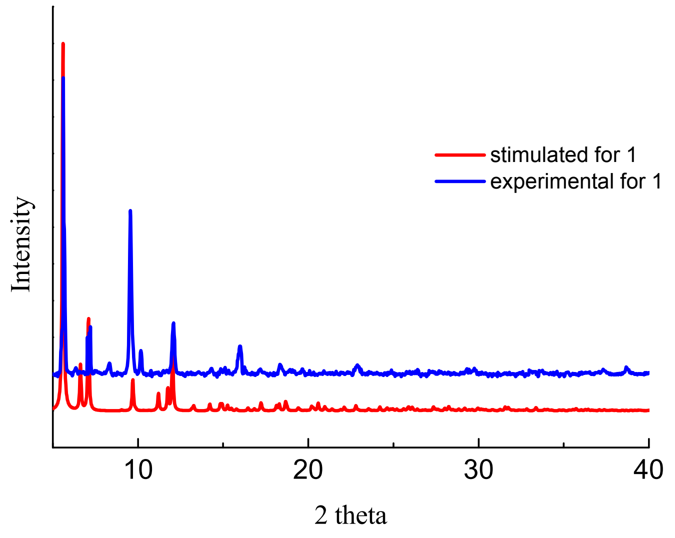

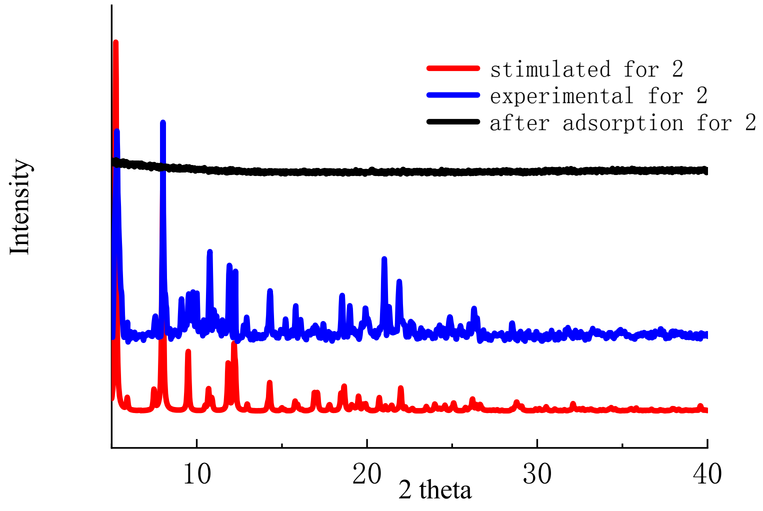

Figure S1. XRD pattern of **1-2**

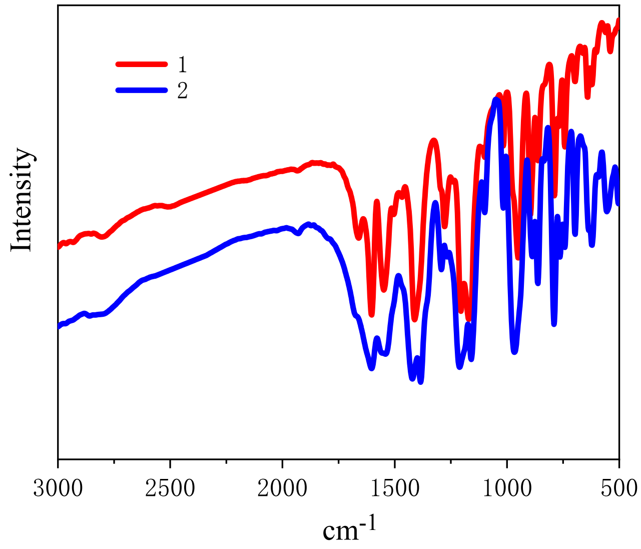

Figure S2. IR spectra of 1 and 2

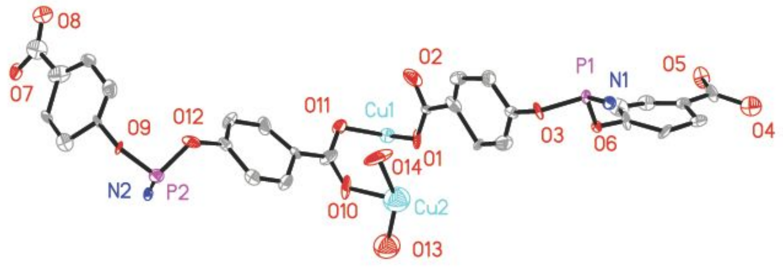

Figure S3. ORTEP drawing (30% probability) of the asymmetric unit of **1** along with the atom numbering scheme. (H atoms were omitted for clarity)

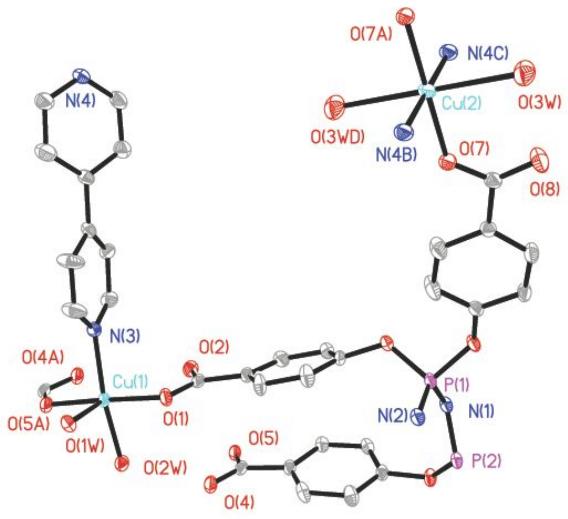

Figure S4. ORTEP drawing (30% probability) of the asymmetric unit of **1** along with the atom numbering scheme. (H atoms were omitted for clarity). Symmetric code: A, -x+2, -y, -z+1; B, x-1/2, -y+1/2, z-1/2; C, -x+1, y, -z+3/2 ; D, -x+1/2, -y+1/2, -z+1.
